# 2D Quantitative Imaging of Magnetic Nanoparticles by an AC Biosusceptometry Based Scanning Approach and Inverse Problem

**DOI:** 10.3390/s21217063

**Published:** 2021-10-25

**Authors:** Gabriel Gustavo de Albuquerque Biasotti, Andre Gonçalves Próspero, Marcelo Dante Tacconi Alvarez, Maik Liebl, Leonardo Antonio Pinto, Guilherme Augusto Soares, Andris Figueiroa Bakuzis, Oswaldo Baffa, Frank Wiekhorst, José Ricardo de Arruda Miranda

**Affiliations:** 1 Biosciences Institute of Botucatu, São Paulo State University, Botucatu 18618-689, São Paulo, Brazil; gabriel.biasotti@unesp.br (G.G.d.A.B.); marcelo.tacconi@unesp.br (M.D.T.A.); leonardo.antonio@unesp.br (L.A.P.); guilherme.soares@unesp.br (G.A.S.); jose.r.miranda@unesp.br (J.R.d.A.M.); 2 Physikalisch-Technische Bundesanstalt, Abbestraße 2–12, D-10587 Berlin, Germany; maik.liebl@ptb.de (M.L.); Frank.Wiekhorst@ptb.de (F.W.); 3 Institute of Physics, Federal University of Goiás, Goiânia 74690-900, Brazil; bakuzis@ufg.br; 4 Faculty of Philosophy, Sciences and Letters at Ribeirão Preto, University of São Paulo, Ribeirão Preto 14040-900, Sao Paulo, Brazil; baffa@usp.br

**Keywords:** magnetic nanoparticles, quantitative imaging, AC Biosusceptometry, inverse problem

## Abstract

The use of magnetic nanoparticles (MNPs) in biomedical applications requires the quantitative knowledge of their quantitative distribution within the body. AC Biosusceptometry (ACB) is a biomagnetic technique recently employed to detect MNPs in vivo by measuring the MNPs response when exposed to an alternate magnetic field. The ACB technique presents some interesting characteristics: non-invasiveness, low operational cost, high portability, and no need for magnetic shielding. ACB conventional methods until now provided only qualitative information about the MNPs’ mapping in small animals. We present a theoretical model and experimentally demonstrate the feasibility of ACB reconstructing 2D quantitative images of MNPs’ distributions. We employed an ACB single-channel scanning approach, measuring at 361 sensor positions, to reconstruct MNPs’ spatial distributions. For this, we established a discrete forward problem and solved the ACB system’s inverse problem. Thus, we were able to determine the positions and quantities of MNPs in a field of view of $5\times 5\times 1{\;\mathrm{cm}}^{3}$ with good precision and accuracy. The results show the ACB system’s capabilities to reconstruct the quantitative spatial distribution of MNPs with a spatial resolution better than 1 cm, and a sensitivity of 1.17 mg of MNPs fixed in gypsum. These results show the system’s potential for biomedical application of MNPs in several studies, for example, electrochemical-functionalized MNPs for cancer cell targeting, quantitative sensing, and possibly in vivo imaging.

## 1. Introduction

Magnetic nanoparticles (MNPs) present several desirable characteristics for diagnostic and therapeutic applications, where the MNPs can be employed for tumor detection, heat generation, or drug delivery [1,2]. Therefore, techniques capable of detecting and quantifying MNPs’ distribution within specific body regions, such as in tumors and organs, are essential to optimize and evaluate biomedical procedures using MNPs [3,4].

Magnetorelaxometry (MRX), Magnetic Particle Imaging (MPI), and Magnetic Susceptibility Imaging (MSI) are promising techniques proven to detect, quantify, and reconstruct spatial distributions based on the MNPs’ magnetic properties. Each of these techniques is based on a specific property of the MNPs. The MRX relies on the time-dependent relaxation of MNPs after a brief magnetization pulse, by both Brownian and Néel mechanisms, to localize and quantify the MNPs [3]. The MPI modality detects the nonlinear magnetization behavior of the MNPs to track and quantify MNPs [5]. The MSI system explores the nonlinear [6] or linear [7] magnetic susceptibility to perform quantitative imaging of MNPs. Although each of these techniques employ a different physical MNPs properties, they all use magnetic field sensors to detect the MNPs’ response after or during exposure to a magnetizing field, which also makes the results of this work of interest for these methodologies. Furthermore, by using multiple magnetizing fields and magnetic field sensors, the quantitative spatial MNPs’ distribution can be reconstructed by solving the ill-posed inverse problem [3,5].

Alternate Current Biosusceptometry (ACB) is a gradiometric coil-based system in which inhomogeneous alternate magnetic fields magnetize the MNPs. Simultaneously, induction coils detect the dynamic magnetic response of the MNPs [8]. Thus, similarly to the MRX, MPI and MSI, the ACB system employs a specific MNP magnetic property (AC susceptibility) to direct detect the MNPs’ sample. The ACB system is extensively employed in gastrointestinal [9,10,11] and pharmaceutical [12,13,14] studies because of its characteristics. Our group recently introduced an ACB single-channel system as a new tool to detect MNPs in vivo, and also to quantify the MNPs’ biodistribution and accumulation in ex vivo and in vitro samples [15,16,17,18,19,20]. However, despite a reasonable FOV (field of view), temporal resolution and sensitivity, the assessment of the quantitative MNPs’ spatial distribution is still pending [17].

More specifically, Corá et al. (2005) applied ACB to evaluate the magnetic tablets’ disintegration process in the human stomach by images [12]. The methodology employed was able to assess the disintegration process of the tablets, and the images obtained were based in voltage values detected in different coil positions, and consequently were not able to provide information about the mass of magnetic material in each position. The same methodology using an ACB system with an array of detection coils was employed to map MNPs’ biodistribution in vivo, using rats [17]. This methodology could assess the MNPs’ pharmacokinetic profile in both the bloodstream and liver, but again was not able to provide quantitative imaging of the MNPs. Moreover, previous studies showed the potential of the ACB system to perform quantitative ex vivo biodistribution studies of MNPs employing organs and cells samples with both position and volume fixed (1D quantitative signals) [15,16,19,20]. Therefore, it is desired to both assess the ACB system’s abilities for imaging and quantitative analysis, and to increase its spatial resolution, to perform MNP quantitative imaging of tablets, organs, and anatomical structures, which can be used in pharmaceutical and physiological studies [12,16,17,21,22]. Such an achievement can create new possibilities of ACB applications in biomedical studies involving MNPs. It is noteworthy that, compared with other techniques, the ACB system does not demand magnetic shielding, presents good portability, and has relatively lower costs, while still providing a lower sensitivity (particularly regarding depth) and spatial resolution.

Similar to MRX, MPI, and MSI, by solving an ill-posed inverse problem it is possible to achieve the quantitative spatial reconstruction of MNPs’ distribution using the ACB system. Therefore, solving the ACB inverse problem, and developing and using a forward model that links the MNPs’ mass in each voxel to the ACB signal measured by each sensor in different positions, enables the quantitative spatial reconstruction of MNPs’ distributions [23].

Firstly, Bastuscheck and Williamson (1995) proposed a formulation for integrating the magnetic flux detected by SQUID (Superconducting Quantum Interference Device) sensors from extended magnetic sources [24]. Although the model enabled the calculations of an induced signal generated by a magnetic sample, with magnetic susceptibility χ and magnetized by an external magnetizing field, the model was based on continuous volumetric integrals, which hampers the model applicability in terms of determining both the mass and position of the magnetic source at the same time. Furthermore, depending on the problem symmetry, the calculation of magnetic fields by the Biot–Savart law may be not straightforward. The calculations are commonly performed by solving elliptic integrals, which can involve a significant computational cost and extensive time. More recently, Hanson and Hirshman (2002) introduced a simple numerical method of the Biot–Savart law, enabling faster calculations of magnetic fields produced by filamentary segments [25]. Here, we adapted the Batuscheck and Williamson formulation, and employed the Hanson and Hirshman method and Faraday’s law, to establish a forward model for the ACB system, which enabled both the discretization of the problem (i.e., establishing voxels) and linking the MNPs’ mass in each voxel to the ACB signal acquired. By minimizing the least square differences between model and measurements, the most probable locations and quantities of MNPs causing the detected signals are found as the solution of the inverse problem [23].

Due to the problem characteristics (i.e., low information and a high number of possible solutions), the problem is ill-conditioned. Several methods have been proposed to improve the conditioning of inverse problems. Liebl and co-workers (2014) experimentally demonstrated that the use of multiple inhomogeneous magnetizing fields can improve the inverse problem conditioning in MRX, thus presenting better results than conventional homogeneous fields [26]. Moreover, Baumgarten and Haueisen (2010) showed that including temporal information in the MRX model also improved the inverse problem conditioning [27]. In general, the addition of information in the forward model (e.g., increasing the number of equations in the problem) improves the system matrix conditioning and stabilizes the solution of the inverse problem [26], allowing more accurate quantifications and a higher spatial resolution to be obtained.

Previously, Jaufenthaler et al. (2020) solved an ill-conditioned inverse problem to perform quantitative 2D MRX imaging of MNPs by employing optically pumped magnetometers (OPMs) [4]. The group showed that, by using OPMs and solving an inverse problem, it is possible to develop a portable MNP imaging setup. In this study, we aimed to develop an ACB setup for 2D quantitative imaging of MNP distributions, which is envisioned for ex vivo quantitative imaging of MNP distributions in organs and tissues. For instance, Wiekhorst et al. (2012) dissected pig lungs into smaller measurable pieces to quantify the MNP distribution in the whole organ using MRX [3]. Employing the presented setup, it is possible to obtain this quantitative information in a non-destructive and automatized manner. At the same time, the knowledge gained in terms of technology and methodology will serve as a starting point to develop advanced imaging setups towards 3D and in vivo quantitative MNP imaging by ACB.

Here, we aimed to develop for the first time an ACB scanning approach, and a discrete ACB forward model and its inverse solution, to quantitatively image 2D distributions of MNPs. This methodology is able to determine the MNPs’ mass and position with good precision and accuracy, and much better spatial resolution (at least 1 cm) than in the previous ACB studies. The scanning approach allows obtaining a high sensitivity (reaching a minimum of 1.17 mg of MNPs) due to short sensor-to-source and excitation coil-to-source distances (0.5 cm) over the entire FOV and an adjustable spatial resolution that depends on the defined scan grid. Compared to a multi-channel ACB imaging approach, the required acquisition time is much higher. However, this drawback is not critical for ex vivo samples. Gypsum phantoms with different concentrations of MNPs were considered to study the ability of the ACB system to perform quantitative imaging of MNPs. In addition, we compared both experimental and calculated system matrices for solving the inverse problem and reconstructing the MNP distributions in the phantoms. Additionally, the results presented here are also applicable to other ACB imaging modalities and will be of great value for the development of new imaging setups.

## 2. Materials and Methods

### 2.1. ACB Single-Channel System

**Figure 1:**
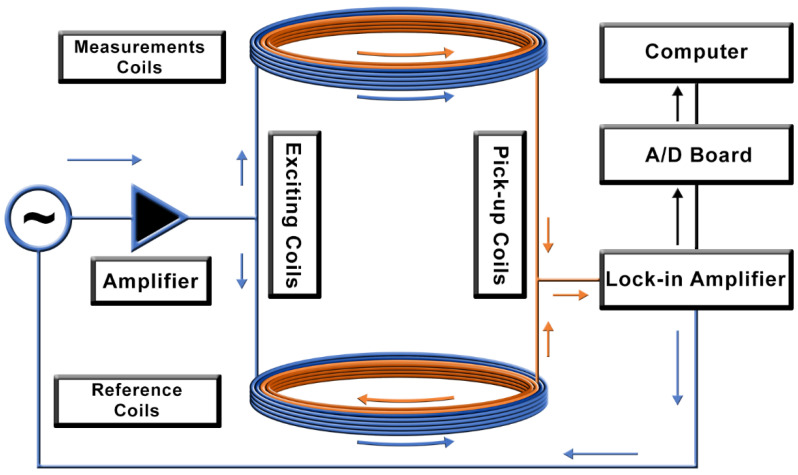
Schematic diagram of the Alternate Current Biosusceptometry (ACB) system. The blue arrows indicate the excitation windings of the coils, and the orange arrows indicate the pick-up windings of the coils.

The ACB single-channel system consists of two pairs of excitation and pick-up coils, in which the detection coils are connected in a first-order gradiometric arrangement, as shown in Figure 1. The system works as a double magnetic flux transformer with an air core. It can be divided into two sub-systems: the measurement and the reference system. Both sub-systems are composed of one excitation and one pick-up coil, with a coaxial arrangement where the inner coil is the pick-up coil. In practice, the measurement system is positioned near the sample, and is responsible for exciting and detecting the MNPs. The reference system is placed far from the sample because of its long baseline (i.e., it is not sensitive and does not contribute to the magnetization of MNPs), detecting the environmental noise and the exciting magnetic field contributions, which will be subtracted by the gradiometric arrangement. Theoretically, no signal arises in the pick-up coils when no MNPs are near the measurement system. In practice, the system is balanced to reach a common mode rejection of at least $1.2\times 10^{-4}$. When an MNP sample is near the sensor, there is an imbalance in the total system’s magnetic flux, and an electric signal is generated in the pick-up coils.

We used a lock-in and an audio amplifier to apply a current in the excitation coils, which will generate the excitation field for the magnetizing of the MNPs. As a result of the pick-up coils’ gradiometric configuration, the lock-in amplifier offers signal pre-processing and reduces environmental noise, removing the need for magnetic shielding.

We built the single-channel sensor (Figure 1) with pick-up coils with an internal diameter of 10.14 mm, an external diameter of 11.84 mm, and 450 turns (AWG 36), and excitation coils with an inner diameter of 13.50 mm, an external diameter of 22.3 mm, and 150 turns (AWG 24). The coils were wound in a multi-layer’s solenoid configuration with a width of 10 mm, and a baseline of 15 cm between the pair of the measurement and reference coils.

### 2.2. ACB System Forward Model and Reconstruction

According to Bastuscheck and Williamson (1995), the magnetic flux generated in the pick-up coils by an extensive magnetized source can be described as:

(1) $$\phi_{s}=\frac{1}{\mu_{0}I_{r}}\int_{V}\chi \left(V,r\right)B_{a}\left(V\right)\cdot B_{r}\left(V\right)dV,$$

where $\phi_{s}$ is the magnetic flux generated in the pick-up coil, $\mu_{0}$ is the magnetic permeability in a vacuum, 𝐼𝑟 is the pick-up coil reciprocal current, 𝜒 the material’s magnetic susceptibility, $B_{a}$ the excitation field, and $B_{r}$ the reciprocal field [24]. This model was conveniently built using the reciprocity principle, where we can interchange detector and source positions to compute the induced signal. Using the reciprocity principle allows application of the numerically efficient expression of the Biot–Savart law by Hanson and Hirshman (2002) for both excitation and sensing [25]. By Hanson and Hirshman’s model, the magnetic flux density, at the center of *k*-th voxel, produced by a coil, is given by

(2) $$B_{k}=\frac{\mu_{0}I_{c}}{4\pi }\sum_{i}\frac{\left(||p_{1,i,k}||+||p_{2,i,k}||\right)\left(p_{1,i,k}\times p_{2,i,k}\right)}{||p_{1,i,k}||||p_{2,i,k}||\left(||p_{1,i,k}||||p_{2,i,k}||+p_{1,i,k}\cdot p_{2,i,k}\right)},$$

where $p_{1,i,k}$ and $p_{2,i,k}$ are the vectors that respectively connect the start point and the final point of *i*-th segment of the coil’s wire to center of the *k*-th voxel. Using Equation (2), each turn of the coil can be approximated by 36 rectilinear segments to maintain the calculations errors below 1% [26].

For quantitative 2D imaging, a discrete forward model for ACB is necessary. Thus, we discretize the problem, and consequently, the FOV is composed of *K* voxels. Hence, the voltage induced in the pick-up coil, generated by an MNPs mass (${Χ}_{MNP,k}$) in the *k*-th voxel, can be calculated by Faraday’s law and has a final form as:

(3) $$V_{n,\;\;k}=-\frac{d}{dt}\left[\frac{\mu_{0}}{I_{r}}.\left(H_{r,n,k}\cdot H_{e,n,k}\right).\chi \left(\omega \right).{Χ}_{MNP,k}\right],$$

where $V_{n,k}$ is the voltage induced in the pick-up coil,$\;H_{r,n,k}$ and $H_{e,n,k}$ are the reciprocal and the magnetizing field, respectively, at the *k*-th voxel with the *n*-th position sensor. Note that in our ACB setup, the MNPs are magnetized by an alternate magnetic field with a constant frequency. Thus, the frequency-dependent mass susceptibility χ(ω) of the MNPs becomes constant in our model. Equation (3) can be extended to *N* sensor positions and *K* voxels. Using matrix notation and separating the geometric parameter and MNPs’ properties from the MNPs’ mass within each voxel, we obtain the following expression:

(4) $$V=\sum_{k}L.{Χ}_{MNP,k}=L\cdot X_{MNP},$$

where V is a vector containing the induced voltages in each sensor, L is the sensitivity matrix of the system, which comprises both geometric parameters and MNPs’ properties, and contains all the sensitivity coefficients that link the mass of MNPs in the *k*-th voxel to a measurement signal in the sensor at the *n*-th position, and $X_{MNP}$ is the vector that represents the MNP mass in each voxel. It is important to point out that the sensitivity matrix L can be determined a priori by calculations or measurements, as described later. Additionally, considering a system with *N* sensors (positions) and *K* voxels will result in a matrix $L\in {\mathbb{R}}^{N\times K}$.

The inverse problem in ACB is often ill-posed; thus, multiple MNP distributions may explain the measurement data. To find the most probable MNP distribution in our imaging problem, we applied linear optimization techniques, such as minimum-norm estimation, employing the Moore–Penrose pseudo-inverse ($L^{+}=\left(L^{T}L\right){\;}^{-1}L^{T}$) calculated by Truncated Singular Value Decomposition (TSVD), as has been shown to be feasible for similar imaging problems [26,28]:

(5) $${\hat{X}}_{MNP}=L^{+}\cdot V,$$

where ${\hat{X}}_{MNP}$ is a vector containing the estimated MNPs’ mass in each voxel. This work determined a truncate limit of 5% for the experimental matrix and 1% for the calculated matrix.

### 2.3. MNP Phantom Development

We employed custom-made MNPs, with a manganese ferrite core (MnFe2O4), synthesized by the coprecipitation method as described previously [29], with a mean diameter of 15 ± 5 nm measured using Transmission Electron Microscopy, and saturation of magnetization of 49.4 emu/g, measured by Vibrating Sample Magnetometry. Fe and Mn content were around 74.4% and 25.6%, respectively, as shown previously. Further details of the MNPs’ synthesis and characterization can be found in [18,30].

Different quantities of MNPs were immobilized in gypsum for the experimental measurements. Thus, the MNPs’ Brownian relaxation was suppressed and Néel relaxation was the dominant relaxation mechanism (so that the effective relaxation time equal approximately to apply the Néel relaxation time $\tau_{eff}\;\approx \;\tau_{N}$). By drying the MNP gypsum mixture in defined containers, cubic MNP phantoms with a volume of $1{\;\mathrm{cm}}^{3}$ were constructed. Five cubes with 0.43, 1.61, 6.77, 9.68, 12.38, and 12.88 mg (A, B, C, D, E1, and E2, respectively) of MNPs were constructed for the 1 cube distribution sensibility test and the 2 cubes distribution spatial resolution test. Moreover, were made 10 similar cubes with an MNP mass of approximately 6 mg to perform several cubes’ distributions in the geometry of the letters: *B*, *I*, *O*, *M*, *A* and *G*.

### 2.4. Experimental and Calculated Sensitivity Matrices Acquisition

#### 2.4.1. Experimental Sensitivity Matrix ($L_{exp}$)

**Figure 2:**
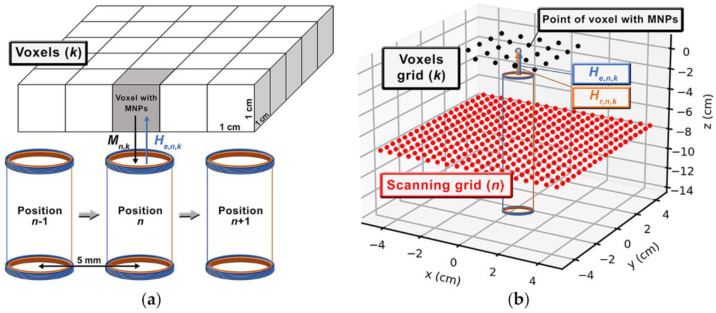
Schemes of sensitivity matrix (***L***) acquisition using: (**a**) the experimental and (**b**) calculated ACB single-channel scanning approach.

To perform quantitative imaging of MNP distributions, we employed a scanning approach, performed by a 2D CNC (Computed Numeric Control) stage, which moved the sensor in a $9\times 9{\;\mathrm{cm}}^{2}$ grid with a step of 0.5 cm. The scanning approach represents N=361 sensor positions in the forward model, used to acquire the MNP signals in $5\times 5\times 1{\;\mathrm{cm}}^{3}$ FOV, with $1{\;\mathrm{cm}}^{3}$ voxels and centralized with a sensor grid. Figure 2 shows an example of the ACB single-channel scanning of an MNP distribution in a 2D voxel grid.

During experimental signal acquisition, a lock-in amplifier and an audio amplifier applied a root-mean-square (RMS) current of 500 mA oscillating at 10 kHz in the excitation coils. The same lock-in measured the voltage ($V_{n}$) induced in the detection coils for each of the *N* sensor positions.

We built a custom-made controlling system to perform all measurements in the LabView^®^ and Mach3Mill^®^ environment. Briefly, after being converted to the voltage RMS value by the lock-in amplifier (with a time constant of 1 ms), the signal was recorded at a sampling frequency of 20 Hz using an A/D acquisition board (National Instruments–NIDAQPad 6015). Simultaneously, the CNC stage moved the sensor, stopping 0.5 s for the voltage acquisition in each position. Furthermore, the CNC stage generated a clock signal with a width of 0.5 s (acquisition period in each position) collected in the same A/D board.

The experimental sensitivity matrix ($L_{exp}$) was built performing 25 scans, in which the sensor was moved in each of the 361 positions (Figure 2a). Each scan was performed with the cube D1 positioned in one of the K=25 voxels. Therefore, we obtained a sensitivity matrix $L_{exp}\in {\mathbb{R}}^{361\times 25}$.

Furthermore, we built an experimental sensitivity map that summarized each sensor position’s contributions in each voxel. All measurements were made with a distance of 5 mm between the sensor and cube surfaces.

#### 2.4.2. Calculated Sensitivity Matrix ($L_{cal}$)

Similarly, the calculated sensitivity matrix $L_{cal}$ was constructed through the computational simulation of the ACB system, as shown in Figure 2b.

Because the magnetic flux calculation does not have a simple analytic solution, especially in non-symmetric situations, the numerical model introduced in Equation (2) was applied to determine $H_{r,n,k}$ and $H_{e,n,k}$. Two considerations were made for using this method: (i) each turn of the coils was polygonally composed of 36 segments, and (ii) the magnetizing field at one point was the sum of fields from all segments.

For the calculations, the real values of the current amplitude (0.5 A) and frequency (10 kHz) were considered. Regarding $L_{exp}$, $L_{cal}$ was built with the virtual sensor positioned in each of the 361 positions. A single voxel with MNP was considered to occupy a different virtual voxel in each scanning, thus resulting in $L_{cal}\in {\mathbb{R}}^{361\times 25}$. All the calculations were conducted considering a 5 mm distance between the voxel and sensor surfaces. Once again, we built a sensitivity map using $L_{cal}$ by summing the signals of all sensor positions for each cube position in a voxel.

### 2.5. Experiments and Quantifications

Sensitivity analysis measurements were assessed scanning five MNPs’ cubes separately (cubes A, B, C, D, and E2), where each cube was positioned in the central voxel of the FOV. We further scanned two similar cubes (E1 and E2), placing the cubes with distances between the cube’s surfaces of 0, 1, and 3 cm along the FOV’s central line. Using the reconstructed images’ correlation and quantification absolute relative error, as detailed below, we determined a threshold distance (spatial resolution in centimetric voxel) above which the system can quantitatively separate the two cubes.

Furthermore, similar cubes of 6 mg were placed and scanned in the FOV to compose *B*, *I*, *O*, *M*, *A*, *G* letters. These data were used to check the method performance in reconstructing three or more cubes at the same time, and the influence of the number of scanning points in the reconstructed image, and to qualitatively compare the voltage maps (no inverse problem solution) with the quantitative images (inverse problem solution).

To assess the influence of the number of scanning points (i.e., the number of coils in the forward problem), the letters’ distributions were reconstructed and evaluated with a horizontal and vertical grid step of 0.5, 1.0, and 1.5 cm, to obtain 361, 100, and 49 grid points, respectively. All of these matrices presented the same voxel, scanning grid, and FOV geometry and sizes.

For each data set, we applied a minimum norm reconstruction based on $L_{exp}$ and $L_{cal}$, respectively. For imaging quality evaluation, all reconstructed distributions ${\hat{X}}_{MNP}$ were correlated with the nominal distribution $X_{nom}$ using the Pearson Correlation Coefficient (*CC*) [31,32]:

(6) $$CC=\left|\frac{\sum_{k}\left({\hat{X}}_{MNP,k}-mean\left({\hat{X}}_{MNP}\right)\right).\left(X_{nom,k}-mean\left(X_{nom}\right)\right)}{\sqrt{\sum_{k}{\left({\hat{X}}_{MNP,k}-mean\left({\hat{X}}_{MNP}\right)\right)}^{2}}.\sqrt{\sum_{k}{\left(X_{nom,k}-mean\left(X_{nom}\right)\right)}^{2}}}\right|$$

where CC=1 is obtained when nominal and reconstructed distribution are equal, and CC=0 when there is no statistical similarity between both distributions. For quantification precision assessments, the absolute relative MNPs’ mass difference, $X_{diff}$, was calculated by comparing the sum of all voxel values in the reconstructed image and the nominal distribution by Equation (7):

(7) $$X_{diff}=\left|\frac{\sum_{k}{\hat{X}}_{MNP,k}}{\sum_{k}X_{nom,k}}-1\right|\cdot 100\%$$

We also studied the dependence of the CC and $X_{diff}$ as a function of the MNPs’ mass to evaluate the impact of the signal-to-noise ratio (SNR) on the imaging quality.

### 2.6. Calculated Voltage Vectors

To evaluate the system performance in reconstructing smaller voxels, we calculated a L matrix with 361 scanning points in a FOV of $5\times 5\times 1{\;\mathrm{cm}}^{3}$ and reduced the voxel length to $0.5\times 0.5\times 1\;{\mathrm{cm}}^{3}$, for a total of 100 voxels. The calculated signal was obtained by solving the forward problem (Equation (4)) and adding noise based on real noise spectra, as proposed by Coene et al. (2014) [33]. The calculated signals were obtained from a distribution of bar phantoms with $5\times 0.5\times 1{\;\mathrm{cm}}^{3}$ and 50 mg of MNPs. Two bars were positioned with distances between the bars’ surfaces of 0, 5 and 10 mm. In addition, four bars were crossed to qualitatively evaluate the spatial resolution. Furthermore, one bar was positioned in the central line of the FOV, and the line spread function (LSF) was obtained to quantitatively determine the spatial resolution by the full width at half maximum (FWHM) method [34].

## 3. Results

**Figure 3:**
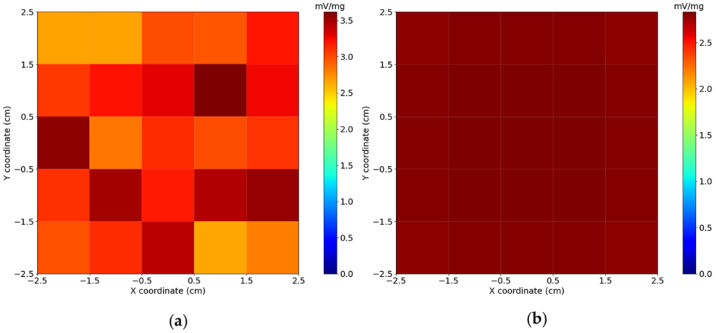
(**a**) Experimental and (**b**) calculated sensitivity maps at a distance of 5 mm between the sensor and voxel surfaces. The Field of View (FOV) has $5\times 5\times 1{\;\mathrm{cm}}^{3}$, and each voxel presents $1{\;\mathrm{cm}}^{3}$. The maps were calculated by summing all the sensor’s signals (normalized by Magnetic Nanoparticles (MNP) mass) for each cube position in a voxel.

The experimental and calculated sensitivity maps are shown in Figure 3. The results showed that the sensitivity map built from $L_{cal}$ presented greater uniformity when compared to the sensitivity map built from $L_{exp}$. A low correlation (CC=0.38) was obtained by comparing both sensitivity maps, which was expected due to the noise inherent in the $L_{exp}$ measurements. Furthermore, the average pixel intensity of the maps is 3.13± 0.28 mV/mg for $L_{exp}$, and 2.81±0.01 mV/mg for $L_{cal}$. These differences in variances and mean values can be related to the real system’s ($L_{exp}$) intrinsic noise, experimental inaccuracies (CNC stage positioning and vibration), and the approximations used in the computational mode.

**Figure 4:**
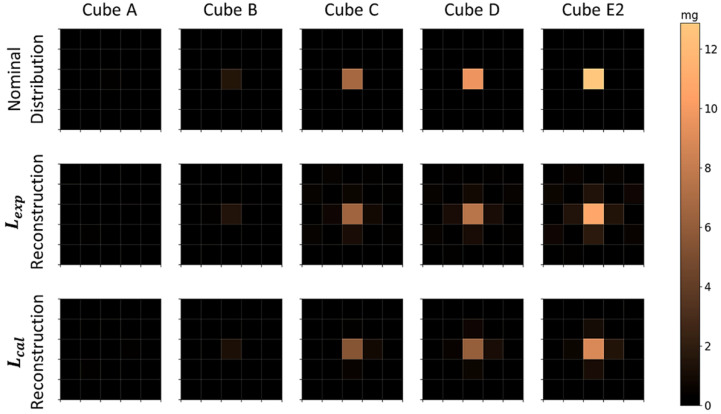
Nominal and reconstructed MNPs’ distribution for a single MNP loaded cube positioned in the center of the FOV. The first row shows the nominal MNP distribution, the second row and the third row show the reconstructions using $L_{exp}$ and $L_{cal}$, respectively. Each column was obtained for an individual MNP cube of different MNP mass (MNP mass increases in ascending order). Physical non-plausible negative MNP masses due to the reconstruction algorithm are not shown.

**Figure 5:**
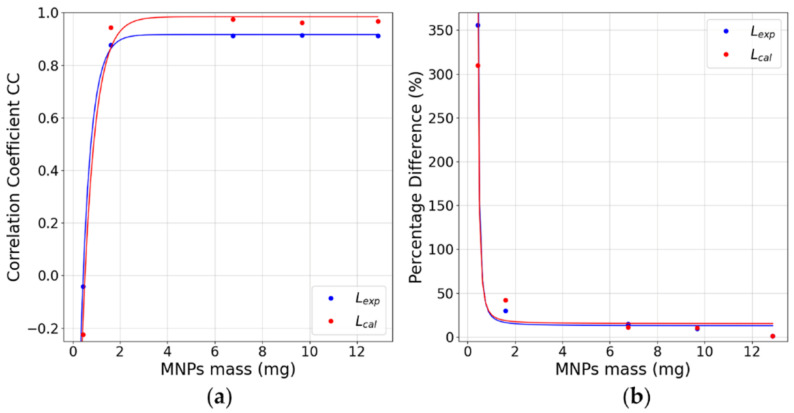
(**a**) *CC* values for reconstructing one cube in the FOV’s center. (**b**) $X_{diff}$ values for the reconstruction of one cube positioned in the FOV’s center. All graphs present the values for all MNP masses used. Fitted curves presented $R^{2}>0.98$.

Nominal and reconstructed MNP distributions for a single MNP loaded cube located in the center of the FOV are shown in Figure 4, where the MNPs’ mass of the cube was varied. For image reconstruction, the Moore–Penrose inverse pseudo-matrices $L_{exp}^{+}$ and $L_{cal}^{+}$ were determined and used to calculate the most probable MNPs’ distributions and quantities using Equation (5). All Pearson correlation coefficients and relative MNPs’ mass differences between the reconstructed and nominal distributions are shown in Table 1. The cubes of higher concentrations (B, C, D, and E2) showed high correlation values and a superior performance in $L_{cal}$ reconstructions. The method did not accurately reconstruct the A cube for both matrices, having high *CC* values, probably related to system noise. Regarding the quantification accuracy, small differences $X_{diff}$ were obtained for cubes C, D, and E2, with no considerable differences between $L_{exp}$ and $L_{cal}$. For cube B, the method showed a moderate decrease in quantification accuracy, and for cube A a severe decrease in quantification was seen with no significant difference between $L_{exp}$ and $L_{cal}$. This can be related to the fact that $L_{cal}$ has lower basal noise, and with this, higher effective reconstruction quality, but reconstructions by both matrices are not sufficient to quantify mass values lower than 1.17 mg (mass value obtained from fitted curves, Figure 5, that result in CC≥0.7 and $X_{diff}\leq 25\%$ simultaneously) of this MNP type. Furthermore, as the scanning area is $9\times 9{\;\mathrm{cm}}^{2}$ and the FOV area is $5\times 5{\;\mathrm{cm}}^{2}$, we did not find differences in the 1 cube reconstruction in the periphery region (not shown).

**Figure 6:**
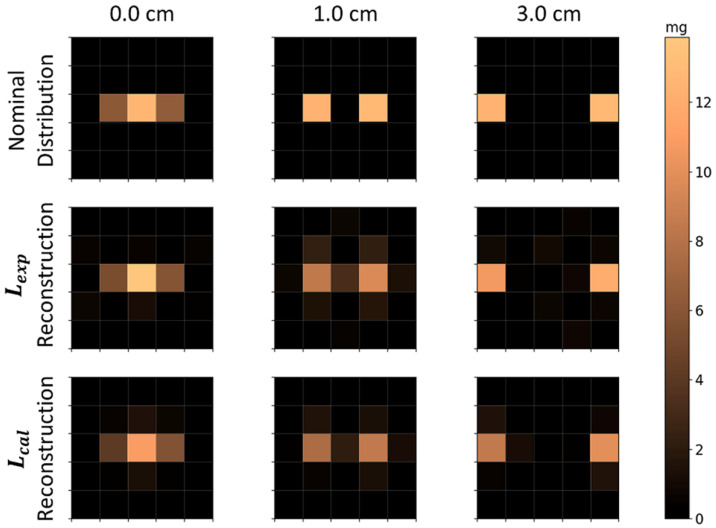
Nominal and reconstructed MNPs’ distributions with two MNP loaded cubes located in the FOV at the same time. The first row is the nominal distributions; the second row shows the reconstructions using $L_{exp}$; and the third row shows reconstructions using $L_{cal}$. The columns indicate the distance between both cubes, e.g., for cube E1 and E2 with the face-to-face distance of 0, 1, and 3 cm.

The nominal and reconstructed MNP distributions for two MNP cubes (E1 and E2), measured simultaneously in the FOV, are presented in Figure 6. Using both $L_{exp}$ and $L_{cal}$, we were able to quantitatively resolve two cubes separated by 1 and 3 cm, where each cube is visible as an individual MNPs’ source in the reconstruction. The correlation coefficients for these distances were high for all cube distances, with a higher performance in $L_{cal}$ reconstruction (Table 2). Moreover, the quantification showed good accuracy with no significant differences between the $L_{exp}$ and $L_{cal}$ methods. For the reconstruction of two cubes placed next to each other (i.e., 0 cm distance), a good correlation (CC>0.98) and good quantification accuracy ($X_{diff}<5\%)$ were obtained. It is worth pointing out that each cube occupies half of the central voxel for 0 cm of distance, summing their contributions in this voxel, and half of the next voxel in the line, contributing with half of their MNPs’ mass in these voxels. These results indicate that was no difference between $L_{exp}$ and $L_{cal}$ results for 2 cubes’ reconstruction, and using this method, the system resolution is at least 1 cm.

**Figure 7:**
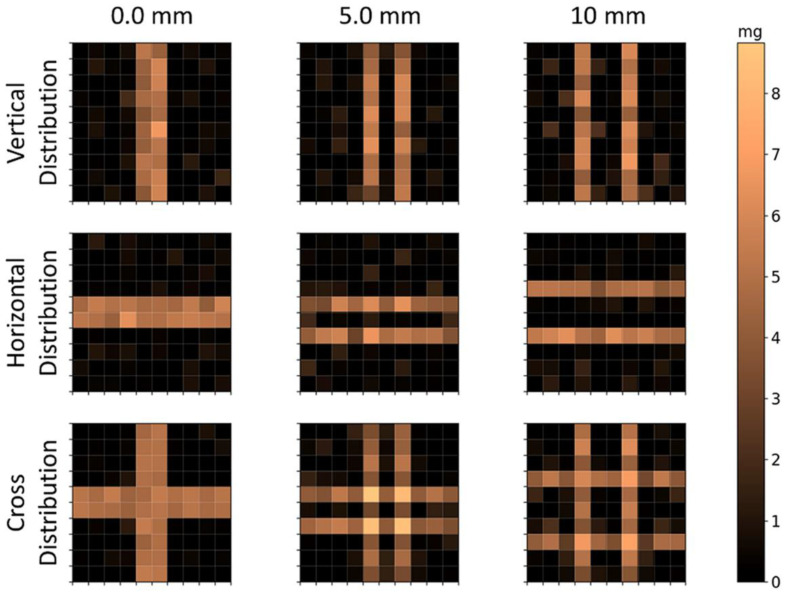
Reconstructed distributions of MNP loaded bars’ calculated signal. Note that lower voxels were used in the same FOV of $5\times 5\times 1{\;\mathrm{cm}}^{3}$, for a total of 100 voxels of $0.5\times 0.5\times 1{\;\mathrm{cm}}^{3}$. The first row shows the vertical distributions; the second row shows the horizontal distributions; and the third row shows the cross distributions. The columns indicate the distance between the bars’ surfaces.

The reconstructions of calculated voltage vectors related to the bar distributions are shown in Figure 7, and the correlation and quantification parameters are shown in Table 3. In these reconstructions, the voxels were reduced from $1\times 1\times 1{\;\mathrm{cm}}^{3}$ to $0.5\times 0.5\times 1{\;\mathrm{cm}}^{3}$, for a total of 100 voxels in the same FOV of $5\times 5\times 1{\;\mathrm{cm}}^{3}$. As $L_{exp}$ and $L_{cal}$ showed similar results in the reconstruction of 25 voxels, these results were obtained only by the calculated method. The *CC* values indicate that is no difference between horizontal and vertical reconstructions. Moreover, the method was able to simultaneously solve the horizontal and vertical bars, with a slight decrease in geometry reconstruction performance. In terms of quantification, all geometries showed high precision ($X_{diff}<1\%)$ with no significant difference between each other. These results show that the scanning method has at least 5 mm of spatial resolution. Using one bar in the vertical disposition, we obtained the LSF of the image to quantitatively evaluate the resolution (image not shown). This reconstructed bar showed a CC of 0.95 and $X_{diff}$ of 1.42%, and the FWHM was 4.59 mm.

**Figure 8:**
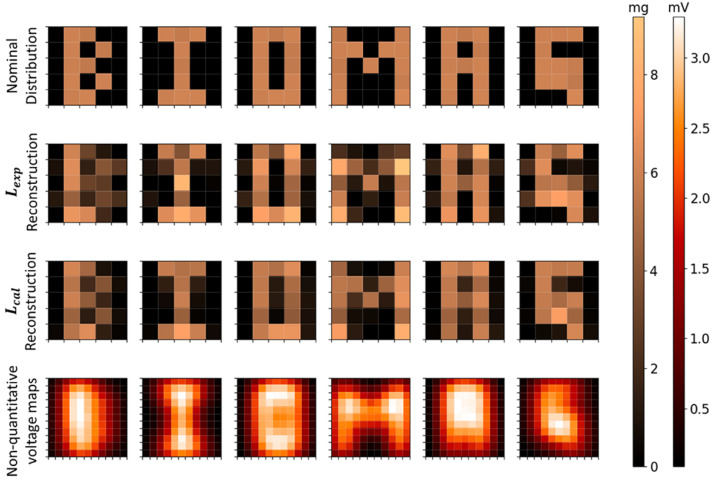
Reconstructed images and voltage maps of MNPs’ distribution in the geometry of letters *B*, *I*, *O*, *M*, *A*, and *G*. The first row shows the nominal distributions; the second row shows the reconstructions using $L_{exp}$; the third row shows the reconstructions using $L_{cal}$; and the fourth row shows the voltage maps.

The reconstruction of the MNPs’ distributions of several cubes arranged to form the letters *B*, *I*, *O*, *M*, *A*, and *G* can be seen in Figure 8. As shown in Table 4, all letters for $L_{exp}$ and $L_{cal}$ presented high correlation values and low relative error in quantification and no significant differences between experimental and calculated methods. The voltage maps of letters’ distributions shown in Figure 8 (fourth row) are based on voltage intensity distributions, detected by the sensor in the respective positions. In comparison to the voltage maps, the inverse problem reconstructions presented a better spatial resolution, because they solved the letters’ geometries, and also allowed a quantitative analysis.

**Figure 9:**
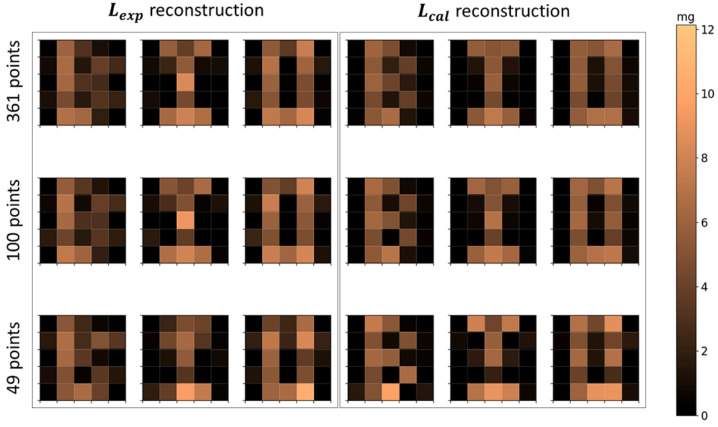
Reconstructed images of MNPs’ distribution in the geometry of letters *B*, *I*, and *O*. The first row shows the reconstructions with 361 scanning points; the second row shows the reconstructions with 100 scanning points; and the third row shows the reconstructions with 49 points. The first three columns use $L_{exp}$ and the last three columns use $L_{cal}$.

The reconstructed letters’ distributions using 361, 100, and 49 scanning points are shown in Figure 9, and the respective quantification parameters are shown in Table 4. The results show that there is not a significant difference between the MNPs’ mass quantification using 361, 100, and 49 scanning point reconstructions. The *CC* values showed a small difference in the quality reconstruction between 361 and 100 points geometries. The 49 points reconstructions showed a considerable reduction in geometry quality in comparison with 361 and 100 points reconstructions. In terms of scanning time, the 361 points scanning took 6 min, the 100 points scanning took 2.5 min, and the 49 points scanning took 1.3 min. Thus, decreasing the number of scanning points may improve the scanning time of ACB, which can improve the method for in vivo applications and the evaluation of physiological dynamic parameters.

## 4. Discussion and Conclusions

There are currently few biomagnetic techniques capable of reconstructing the spatial distribution of MNPs using non-invasive measurements. Among the techniques reported in the literature, those that have drawn attention and show high potential are MPI, MRX, and MSI. MRX has high sensitivity, can detect micrograms of MNPs, and presents a spatial resolution of a few centimeters [3]. MPI can detect and estimate masses of ferromagnetic material at a microgram scale, with millimeter spatial resolution [35,36,37]. Despite having suitable quality for image reconstruction and quantifying the mass of particles with high sensitivity, both MPI and MRX involve high costs. Thus, the development of alternative biomagnetic techniques capable of reconstructing quantitative MNPs’ spatial distributions with low cost, high versatility, and portability, and without the need for electromagnetic shielding, can significantly contribute to scientific research and biomedical applications of MNPs.

Previously, our group showed the effectiveness of the ACB single-channel system in real-time in vivo evaluation of the MNPs’ distribution and retention in the liver [19], its circulation in the bloodstream [18], and its different perfusion profile in healthy and injured kidneys [20] in rats. Some of these papers also employed the ACB single-channel system to quantify the MNPs’ biodistribution in several organs. Others aimed at the MNPs’ cell internalization in ex vivo experiments and also to perform real-time video maps of the qualitative MNPs’ biodistribution [15,17,18,19,20]. Furthermore, recently we showed that ACB can assess the effects of corona protein formation in MNPs with three different coatings, which can be employed in biosensing assays [38]. However, the ACB measurements presented to date did not correlate the pixel intensity with the mass of MNPs.

This study presented the ACB quantitative imaging with in vitro and in silico procedures. We also presented a methodology for an ACB scanning approach, which can increase the problem stability and provide quantitative imaging of the MNPs’ spatial distribution. By building the ACB sensitivity matrix and using Equation (5), derived from the forward model described in Equation (3), it was possible to estimate the MNPs’ mass present in each voxel of the FOV. These procedures may elevate the technique, allowing reconstructions of quantitative images with a better spatial resolution, as shown before for similar MNPs’ imaging problems [17].

Using the present scanning approach, we were able to reconstruct the quantitative MNPs spatial distribution in a FOV of $5\times 5\times 1{\;\mathrm{cm}}^{3}$ at 5 mm from the sensor surface with $1{\;\mathrm{cm}}^{3}$ voxel. In this manner, we obtained a spatial resolution of 1 cm when the SNR is sufficient (MNP masses greater than 1.17 mg), and it was shown that the scanning method has at least $1{\;\mathrm{cm}}^{-1}$ of resolution and may be improved to reach a higher resolution at a millimetric scale. In this way, the present methodology was able to determine the MNPs’ mass and position with good precision, accuracy, and much better spatial resolution (at least 1 cm) than the previous ACB methodologies in the literature, with a relatively high sensitivity [17,21,39]. The measured time for a complete scan was up to approximately 6 min, which can be decreased using other strategies, such as more sensors, continuous scanning, or even by reducing the number of scanning points. In principle, the scanning approach enables an unlimited 2D FOV size. However, increasing the FOV size means increasing the scanning time. It is important to note that as the ACB system is dependent on the MNP’s χ(ω), changing the MNPs, or the excitation field frequency, would increase the sensitivity and reconstruct even lower quantities of MNPs. Here, we used a conventional ACB system with a transformer factor of three, and increasing the transformer factor would reflect a higher system’s sensitivity [40].

Studies involving MRX have demonstrated the technique’s ability to reconstruct tomographic images in a $3\;\times \;6\;\times \;3{\;\mathrm{cm}}^{3}$ FOV with $1{\;\mathrm{cm}}^{3}$ voxels. MRX can achieve a spatial resolution of millimeters for MNPs’ Fe masses higher than a few µg, with an acquisition time of approximately 12 min [26]. In regard to MPI, 3D in vivo scans were performed in a FOV of $20.4\;\times \;12\;\times \;16.8{\;\mathrm{mm}}^{3}$, with submillimeter spatial resolution in horizontal and vertical directions and temporal resolution of a few milliseconds (real-time acquisition). The system showed good sensitivity for tracers in a concentration range of 8 to 45 $\mu \mathrm{mol}\;\left(\mathrm{Fe}\right)l^{-1}\;$ [36]. It is worth mentioning that for MPI, the voxel size depends on the characteristics and concentration of the MNPs [35]. MSI has shown good results in solving 61 voxels with millimetric volume, and by optimizing the system’s features it is possible to solve approximately 108 voxels [39]. Moreover, MSI showed sensitivity up to a distance of 2 cm for milligrams of Fe MNPs [7]. Compared to MRX and MPI, ACB provides similar temporal resolution, but with less sensitivity and spatial resolution and is restricted to 2D reconstructions, at present. However, the ACB system does not demand magnetic shielding and has greater portability. In comparison with MSI, ACB exhibits a similar sensitivity and uses a lower number of voxels for the same sensor to sample distance. Even though MSI and ACB are based on susceptibility measurements, there are substantial instrumental differences between the systems. The ACB is composed of a pair of excitation coils, a gradiometric pair of detection coils, axially arranged, and peripherical electronics optimized for biomedical measurements in vivo [41,42], in vitro [43], and ex vivo [19]. The ACB system’s characteristics enable a simpler electronics utilization and also larger FOV imaging. Future works can also focus on an adapted ACB system to quantitatively monitor MNPs in vivo and in real-time [18,19], and perhaps for 3D reconstructions with greater sensitivity and a large number of voxels.

In this work, we presented a new ACB scanning approach that enabled quantitative imaging of in silico phantoms with precisely known MNP masses. The results demonstrated that the scanning method using the ACB mono channel system can obtain quantitative MNPs’ images with a spatial resolution of at least 1 cm. The system allows quantifying a few mg of MNPs, which could be further improved by changing the coil’s characteristics. In addition, the results obtained through the use of the calculated sensitivity matrix showed that the mathematical-computational model used was adequate, thus allowing new studies based on computational models, given the lower material and temporal cost. These results demonstrate that the ACB system provides quantitative data and can be employed as a tool for MNPs’ quantitative imaging of organs and tissues, non-destructively, with a low cost, high versatility, and portability. This data is unprecedented and applicable to other ACB modalities, and will also impact the development of new ACB setups, aiming for in vivo and 3D imaging, which may help the research field relating to MNPs’ biomedical applications. Moreover, in the future, it is possible to employ the ACB system and MNPs for biosensing, because it is possible to functionalize the MNPs with specific molecules able to react and link with biomolecules of interest. This can lead to new approaches of ACB to evaluate biochemical bonds of MNPs with plasma proteins and cells, especially using electrochemical-functionalized MNPs for cancer cell targeting, quantitative sensing, and possibly in vivo imaging, including 3D images.

## Figures and Tables

**Table 1:** *CC* and $X_{diff}$ estimated values for the reconstructions of one MNP loaded cube in the center of the FOV. Each cube contains a different MNP mass $X_{nom}$.

Cube	$X_{nom}\;(\mathrm{mg})$	${\hat{X}}_{MNP}\;(\mathrm{mg})$		$X_{diff}\;(\%)$		*CC*	
		$L_{exp}$	$L_{cal}$	$L_{exp}$	$L_{cal}$	$L_{exp}$	$L_{cal}$
A	0.43	1.10	0.90	355.76	309.64	0.04	−0.23
B	1.61	1.12	0.93	30.04	42.07	0.88	0.94
C	6.77	7.78	7.52	14.93	11.13	0.91	0.97
D	9.68	8.81	8.67	9.03	10.41	0.91	0.96
E2	12.88	12.98	12.78	0.80	0.72	0.91	0.97

**Table 2:** *CC* and $X_{diff}$ estimated for the ACB reconstructions of two MNP loaded cubes for different distances separating those cubes in the FOV.

Cubes Distance (cm)	$X_{nom}\;(\mathrm{mg})$	${\hat{X}}_{MNP}\;(\mathrm{mg})$		$X_{diff}\;(\%)$		*CC*	
		$L_{exp}$	$L_{cal}$	$L_{exp}$	$L_{cal}$	$L_{exp}$	$L_{cal}$
0	25.26	23.98	24.20	5.05	4.18	0.98	0.98
1	25.26	22.65	23.52	10.33	6.89	0.88	0.95
3	25.26	22.84	24.08	9.57	4.68	0.97	0.98

**Table 3:** CC and $X_{diff}$ values for the ACB reconstructions of the calculated signal of bars’ geometries.

Distribution	$X_{nom}\;(\mathrm{mg})$	${\hat{X}}_{MNP}\;(\mathrm{mg})$			$X_{diff}\;(\%)$			*CC*		
		0 mm	5 mm	10 mm	0 mm	5 mm	10 mm	0 mm	5 mm	10 mm
Vertical	100	99.38	99.61	99.56	0.61	0.39	0.44	0.94	0.94	0.92
Horizontal	100	99.53	99.61	99.53	0.47	0.39	0.47	0.96	0.91	0.95
Cross	180	179.50	179.43	179.57	0.28	0.32	0.24	0.99	0.90	0.92

**Table 4:** CC and $X_{diff}$ values for the ACB reconstructions of calculated signals of bars’ geometries.

Letter	$X_{nom}\;(\mathrm{mg})$	$X_{diff}\;(\%)$						*CC*					
		$L_{exp}$	$L_{e100}$	$L_{e49}$	$L_{cal}$	$L_{c100}$	$L_{c49}$	$L_{exp}$	$L_{e100}$	$L_{e49}$	$L_{cal}$	$L_{c100}$	$L_{c49}$
B	59.05	3.18	3.50	5.42	1.78	1.88	2.93	0.81	0.80	0.78	0.95	0.97	0.92
I	53.47	0.82	1.02	3.08	1.27	1.84	2.04	0.93	0.89	0.78	0.97	0.97	0.90
O	71.14	1.65	1.73	4.07	2.10	2.26	3.36	0.92	0.91	0.85	0.97	0.96	0.88
M	77.57	2.45	1.75	0.12	5.99	6.86	8.49	0.83	0.80	0.79	0.94	0.93	0.80
A	71.14	0.59	0.93	1.85	2.63	2.71	3.90	0.93	0.92	0.86	0.97	0.95	0.87
G	65.87	0.70	0.10	1.47	4.12	4.22	4.27	0.92	0.90	0.85	0.98	0.96	0.90

## Data Availability

Not applicable.
